# Primary mediastinal seminoma with azoospermia: case report and review of the literature

**DOI:** 10.3389/fonc.2024.1309803

**Published:** 2024-05-17

**Authors:** Zhiwei Li, Qiqi Zhu, Shaorui Niu, Kaibing Xiao, Zhiyang Xiao, Pang Yang

**Affiliations:** ^1^ Suzhou Kowloon Hospital, Shanghai Jiaotong University School of Medicine, Suzhou, China; ^2^ Department of Urology, The First Affiliated Hospital of Nanchang University, Nanchang, China; ^3^ Department of Urology, The First Hospital of Nanchang, Nanchang, China; ^4^ Department of Intensive Care Unit, The First People’s Hospital of Guangyuan, Guangyuan, China

**Keywords:** mediastinum, seminoma, azoospermia, germ cell tumor, mediastinal tumor

## Abstract

**Introduction:**

Since the first report, primary mediastinal seminoma has a low incidence in the population, and it mainly affects young and middle-aged men, is clinically rare, and accounts for a very small proportion of mediastinal tumors. In this study, we describe the first case of primary mediastinal seminoma with azoospermia and hypothesize that the coexistence of the two disorders may not be a coincidence.

**Case report:**

A 16-year-old man presented with chest tightness and chest pain, a mediastinal mass on chest CT, and abnormal 18F-fluoro-deoxyglucose uptake on a PET-CT scan. By biopsy of the mass, the pathological diagnosis was a primary mediastinal seminoma. Because chemotherapy is included in the treatment of the tumor, the patient underwent sperm freezing before treatment, considering that chemotherapy can affect fertility, but the patient was diagnosed with azoospermia. Finally, the patient underwent tumor resection and postoperative chemotherapy. No tumor recurrence was observed at the current follow-up.

**Conclusion:**

Primary mediastinal seminoma is mainly confirmed by histopathological examination, and surgery and chemoradiotherapy are the current treatments. In patients with mediastinal seminoma or azoospermia, doctors should be aware that the two disorders may coexist, especially in men who have fertility requirements or long-term infertility, and that examination of the mediastinum and semen may lead to unexpected findings in the diagnosis and treatment. For mediastinal germ cell tumors, genetic testing is of great value in the treatment of tumors and the prediction of associated diseases. Future studies exploring the potential correlation between mediastinal seminoma and azoospermia will be prospective.

## Introduction

Primary mediastinal seminoma was first reported by Woolner et al. in 1955. The tumor is an extragonadal malignant germ cell tumor that usually occurs in men aged 15~40 years. The incidence is low, the disease is relatively rare, and primary mediastinal seminoma accounts for approximately 1% ~ 4% of mediastinal tumors (1–3). The pathogenesis of primary mediastinal seminoma is not fully understood, but it may be related to ectopic gonadal tissue during embryonic development, retention and displacement of some germ cells or pluripotent stem cells to the mediastinum, and induction by carcinogenic factors (4–8). At present, there are many reports of a single tumor, but reports of other diseases are still rare. Here, we describe the first case of primary mediastinal seminoma with azoospermia and hypothesize that the coexistence of the two disorders may not be a coincidence.

## Case report

**Figure 1:**
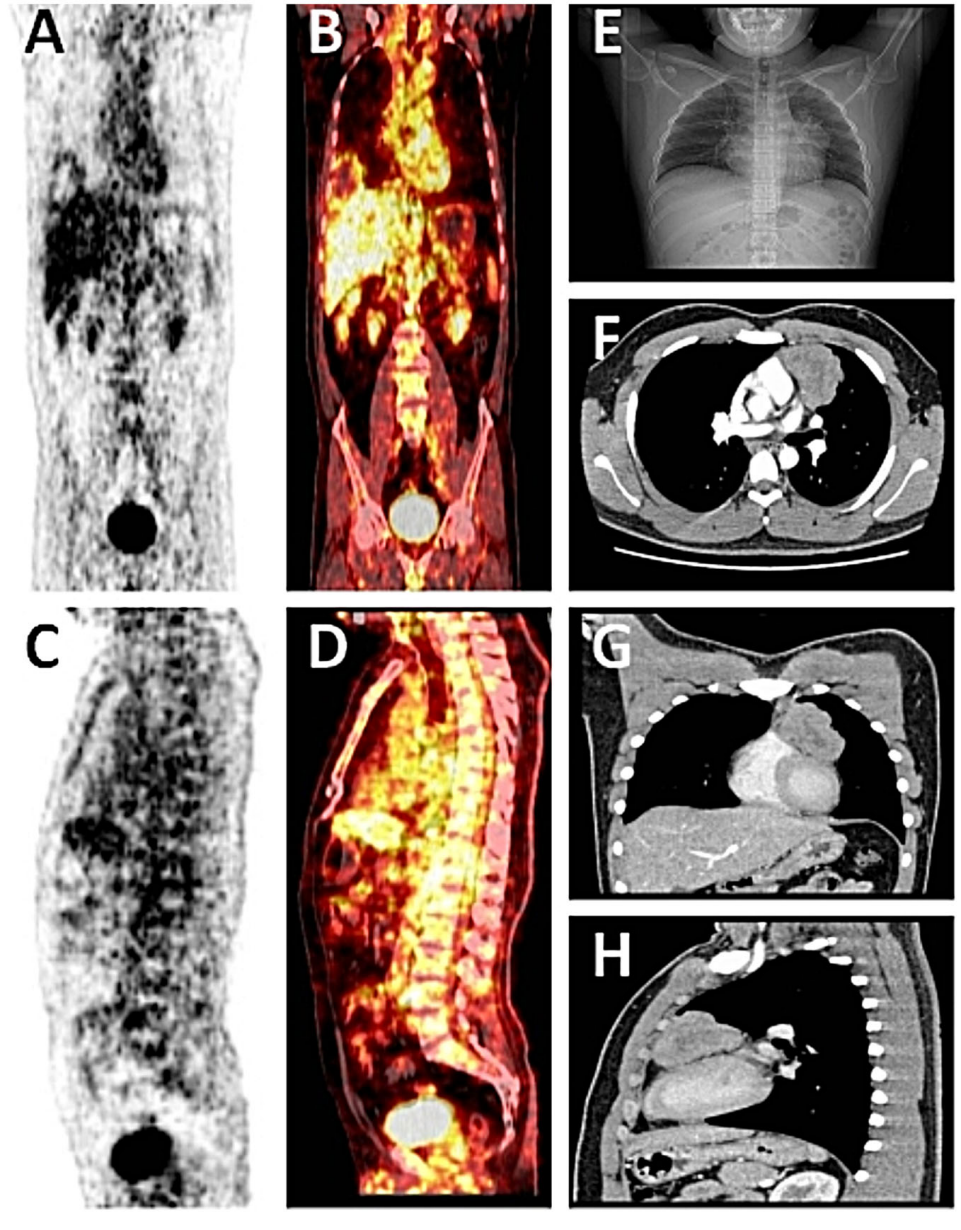
**(A-D)** Whole-body PET-CT showed a left anterior mediastinal soft tissue shadow, an uneven increase in radioactive uptake (SUVmax = 4.5), and no obvious abnormalities in other organ tissue examinations. **(E)** A chest X-ray showed a left anterior mediastinal tumor with illuminated edges. **(F-H)** Chest CT showed a large mass of soft tissue density in the left anterior upper mediastinum, with lobulated edges and uneven internal density, and mild to moderate enhancement of the solid part of the edge on enhanced scanning.

A 16-year-old man presented to the department of thoracic surgery of the hospital with a 2-day history of chest pain and chest tightness. He was previously healthy and did not take any medications. The man’s physical examination was normal; a huge mass in the left anterior mediastinum with a maximum measurement of approximately 58 mm x 46 mm was found on the chest CT examination; and he was hospitalized. In the laboratory analysis results after admission, in addition to uric acid (UA) 528 µmol/l (normal 208–428 µmol/l), urine glucose (UGLU) positive (3+), human chorionic gonadotropin-beta subunit (HCG-β) 313.08 mIU/mL (normal 0–5 mIU/mL), the remaining liver and kidney function, AFP, blood lipids, serum glucose, and systemic inflammatory markers (erythrocyte sedimentation rate and C-reactive protein) were normal. Due to the abnormal HCG-β value, the man underwent a color ultrasound and CT examination of the reproductive system to exclude other diseases, but the results showed that there was no abnormality. To clarify the nature of the mediastinal mass, a needle biopsy was performed on the mass, and the diagnosis was mediastinal seminoma based on the pathological examination combined with immunohistochemistry. Considering that seminoma is a malignant tumor, the possibility of distant metastasis could not be ruled out, so a whole-body PET-CT examination was completed. The results showed that the 18F-fluoro-deoxyglucose metabolism of the left anterior mediastinal mass was increased, and there were no abnormalities in the examination of other organs and tissues (**Figures 1A-D**). To determine the next treatment plan, the hospital organized multidisciplinary treatment (MDT) meetings with the departments of thoracic surgery, urology, oncology, imaging, radiotherapy, and pathology. There are two treatment options that were discussed in the MDT meetings: chemoradiotherapy before surgery or chemoradiotherapy after surgery. Because of the young age of the patient, considering that chemoradiotherapy can affect fertility, it was recommended that the man undergo sperm freezing before treatment. Surprisingly, after rigorous testing by the human sperm bank institution, the man was finally diagnosed with azoospermia. The patient said that he had never had this test before, and the man also told that he had ejaculated spontaneously in the past, but no sperm loss occurred. Because no reproductive disorders were detected on imaging, gonadotropins and lactate dehydrogenase (LDH) were examined. His estradiol (E2) 100.0 pg/ml (normal 11.3–43.2 pg/ml), prolactin (PRL) 15.30 pg/ml (normal 4.04–15.2 pg/ml), follicle stimulating hormone (FSH) < 0.30 mIU/mL (normal 1.5–12.4 mIU/mL), luteinizing hormone (LH) < 0.30 IU/mL (normal 1.7-8.6 mIU/mL), progesterone (PROG), testosterone (T), and LDH levels were normal. Based on the condition, the patient eventually requested a hospital transfer.

**Figure 2:**
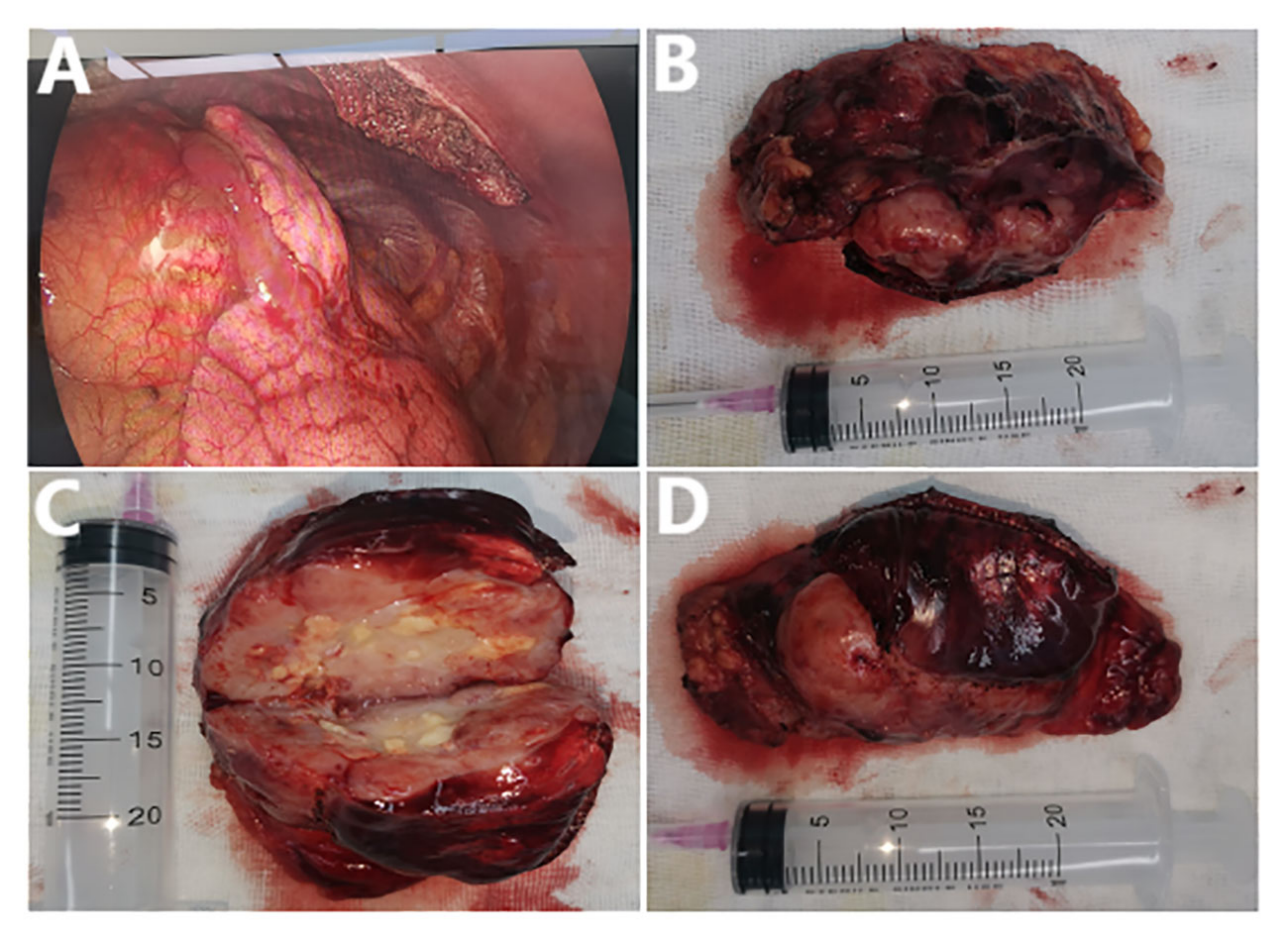
**(A)** The tumor seen during surgery. **(B-D)** The completely removed mediastinal tumor after surgery and the internal conditions after tumor incision.

**Figure 3:**
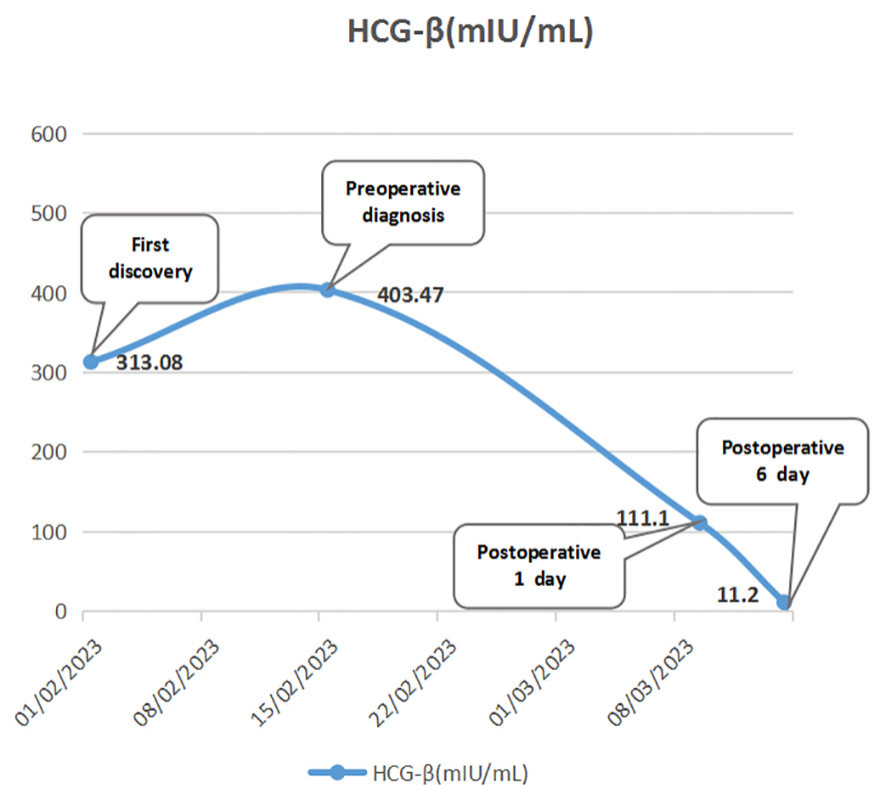
Trends in the patient’s HCG-β levels before and after surgery.

**Figure 4:**
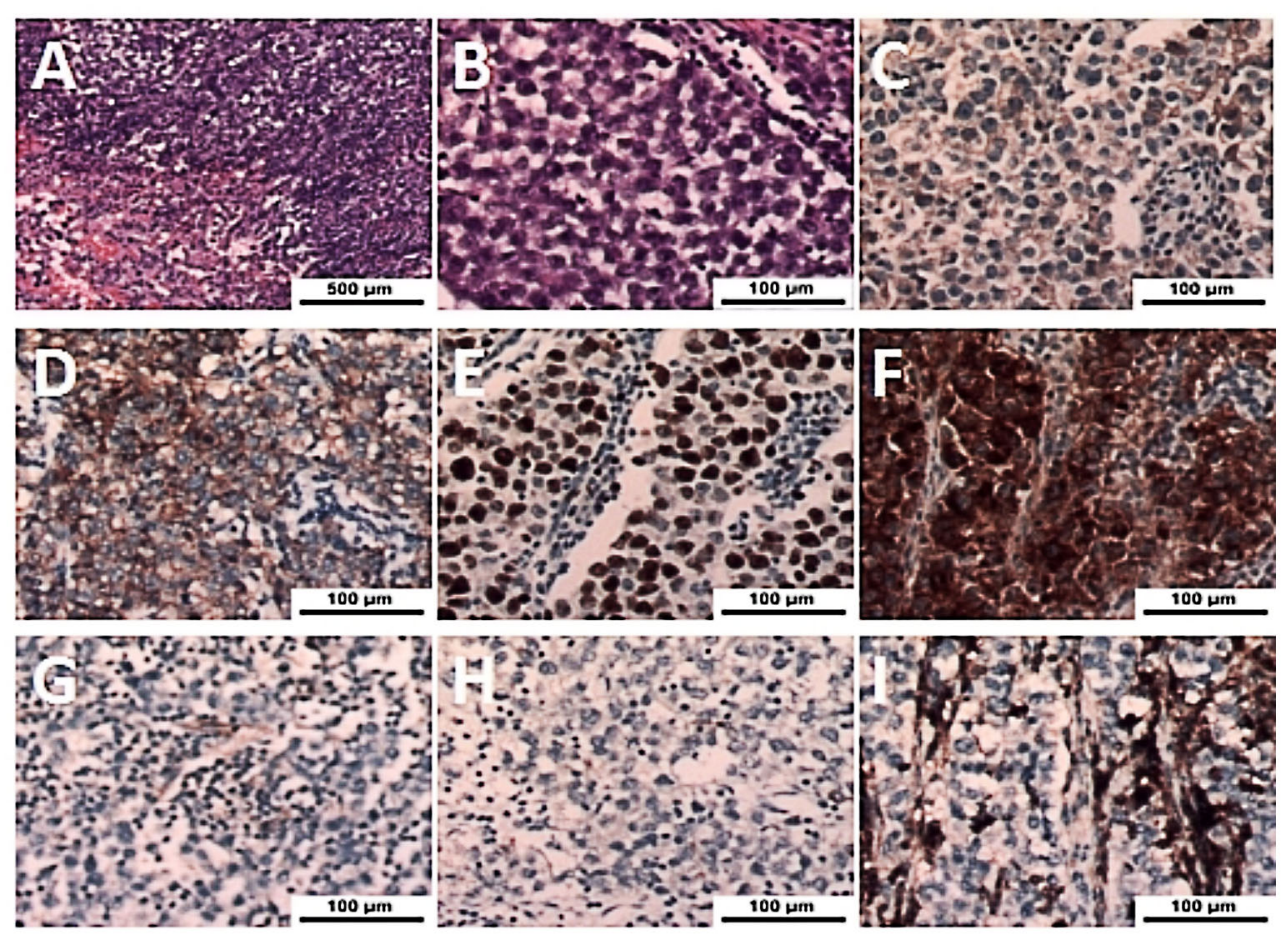
**(A, B)** Histologic features of seminoma with large tumor cells, round, polygonal appearance, deep nuclear staining, round foam nuclei, pathological mitotic images, and lymphocyte infiltration of the interstitium were observed (H&E, magnification x50 and x400). **(C-F)** Immunohistochemistry of tumor cells showed strong expression of AFP (low+), CD117 (+), ki-67 (+), and PLAP (+) (magnification x400). **(G-I)** Immunohistochemistry of tumor cells showed negative results for CD99 (-), CK-pan (-), and LCA (-) (magnification x400).

A week later, the man, accompanied by his parents, came to our hospital for treatment. He told us about the previous diagnosis and treatment, and he decided to undergo surgery and then chemoradiotherapy. After admission, we evaluated and prepared the patient before surgery and found the following: HCG-β 403.47 IU/L, UA 586 µmol/l (normal 155-428 µmol/l), and UGLU positive (2+). A chest X-ray and a chest CT showed a large left anterior mediastinal mass; the maximum measurement was approximately 72 mm x 49 mm, and the remaining preoperative examination results were normal (**Figures 1E-H**). After all the preoperative preparations were complete, we opted for thoracoscopic mediastinal mass resection. During the operation, we observed that the mass was huge and closely adhered to the upper left lung lobe, which did not rule out the possibility that the tumor had invaded the lung lobe (**Figure 2A**). Finally, under the premise of resecting part of the upper left lung lobe, we successfully and completely removed this large tumor. The tumor was actually measured to be approximately 110 mm x 60 mm x 45 mm in size, resembling a kidney, dark red and grayish yellow, hard in texture, and with solid gray-white tissue inside (**Figures 2B-D**). The man remained stable after surgery, and we also compared changes in the laboratory analyses before and after surgery. We found that HCG-β decreased significantly to 111.11 mIU/mL on postoperative day 1 and to 11.2 mIU/mL on postoperative day 6 (**Figure 3**). In addition, LH 0.22 IU/mL (normal 1.24–8.62 mIU/mL), PROG 0.22 nmol/L (normal 0.30–2.60 nmol/L), T 1.75 nmol/L (normal 6.07–27.10 nmol/L), E2, FSH, PRL, and UGLU results are normal. The final pathological diagnosis was mediastinal seminoma involving the lungs (**Figures 4A, B**). Immunocytochemistry (IHC) showed the following: CD117 (+), LCA (-), CK-pan (-), CD99 (-), AFP (low+), PLAP (+), and ki-67 (80%+) (**Figures 4C-I**). A week after the operation, the man recovered well and was discharged from the hospital. During the 3rd week after surgery, the patient had a repeat semen analysis, and the result was still azoospermia. The fluorescence *in situ* hybridization (FISH) method showed t(12p12.1) (KRAS gene locus was abnormal, with KRAS (12p) gene polybody). At 4 weeks postoperatively, the patient started chemotherapy with the EP regimen, specifically etoposide 200 mg + cisplatin 40 mg (d1-d5, 21 days/cycle). At present, the patient has completed 4 cycles of chemotherapy, and no evidence of tumor recurrence has been found thus far.

## Discussion

Seminoma is a malignant germ cell tumor that rarely occurs outside the gonads, and a small number of tumors can occur in extragonadal tissues or organs such as the retroperitoneum, mediastinum, and pineal gland (9, 10). The clinical manifestations of primary mediastinal seminoma are nonspecific, and common symptoms include chest pain, chest tightness, dyspnea, and facial swelling, usually related to tumor size and compression or invasion of adjacent tissues. In asymptomatic patients, smaller tumors are often found incidentally on imaging tests at physical examination (11–13).

In the diagnosis and treatment of primary mediastinal seminoma, misdiagnosis and mistreatment are very likely to occur. Therefore, a comprehensive diagnosis is required based on clinical symptoms, imaging examinations, serum tumor markers, and pathological results. According to the clinical guidelines of the American Society of Clinical Oncology, serum AFP and β-hCG are not only considered important for the qualitative diagnosis and pathological classification, but are also closely related to the evaluation of the efficacy of primary mediastinal seminoma and monitoring of tumor recurrence and metastasis (14, 15). In this case report, our patient showed significant changes in β-hCG values before and after surgery. At present, new diagnostic methods on molecular, immunological, chromosomal, and other aspects are gradually being reported (16–18).

Treatment of primary mediastinal seminoma is mainly chemoradiotherapy combined with surgery to remove the tumor, but the timing of surgery is controversial. Chemoradiotherapy should be carried out first, followed by surgery to remove the residual tumor. It has also been argued that radical tumor resection should be performed before chemotherapy (19–21). In general, the timing of surgical treatment should be determined according to the actual situation of different patients. This tumor is mostly a low to moderately malignant tumor sensitive to chemoradiotherapy; it is also one of the tumors that can be cured by comprehensive treatment of chemoradiotherapy combined with surgery, and early correct diagnosis and standardized treatment are crucial to the prognosis of primary mediastinal seminoma (22, 23).

Azoospermia is the most refractory infertility and is clinically divided into nonobstructive and obstructive azoospermia. Its etiology and pathogenic mechanisms are complex and mostly related to the patient’s environment, chromosomes, sex hormones, and other factors (24–26). The treatment of azoospermia varies greatly due to different causes, and even with the rapid development of assisted reproductive technology, there is no standard treatment, but an individualized treatment plan based on the patient’s etiology can improve the treatment effect (27, 28).

In this paper, we report the first case of primary mediastinal seminoma with azoospermia, and no similar cases have been reported. Azoospermia has been associated with tumors and has a higher incidence of cancer in men with azoospermia (29–31). Although the specific cause of the association between mediastinal seminoma and azoospermia in male adolescents has not been clearly revealed, it may not be a coincidence that the two disorders coexist. In malignant germ cell tumors, mutations in chromosome 12p are most common (32–34). The mutant gene KRAS, which belongs to the Ras gene family and is also a key cause of cancer, is currently confirmed in studies of pancreatic cancer, colorectal cancer, and lung adenocarcinoma but is rarely reported in studies related to mediastinal seminoma and azoospermia. Notably, there is currently no relevant research to clearly explain whether the mutation of the KRAS gene can be used as direct evidence as the cause of these two diseases, but perhaps there will be more cases and studies to explore and confirm this in the future.

There are also limitations to the reporting of this study. First, we did not follow up for a long time, and the time from treatment to the recent follow-up of the cases reported this time was only 7 months. In this case, we have proposed the hypothesis of temporary azoospermia. Transient azoospermia occurs due to a disturbance of gonadal axis hormone levels due to mediastinal seminomas. When hormone levels in the body return to normal after the tumor is treated, azoospermia may resolve on its own. After a long period of follow-up, this hypothesis may be successfully validated. In addition, the patient did not undergo an extraction biopsy of testicular tissue. We were unable to find evidence at the cellular level that the patient’s sperm were hindered in the formation process. Therefore, it is difficult to further clarify the cause of the association between mediastinal seminoma and azoospermia, and it is not possible to provide more effective evidence that KRAS mutations directly affect spermatogenesis.

## Conclusion

Primary mediastinal seminoma is diagnosed mainly based on histopathological examination, and surgery and adjuvant chemoradiotherapy are the current treatments. In patients with mediastinal seminoma or azoospermia, doctors should be aware that the two disorders may coexist, especially in men who have fertility requirements or long-term infertility, and that examination of the mediastinum and semen may lead to unexpected findings in diagnosis and treatment. For mediastinal germ cell tumors, genetic testing is of great value in the treatment of tumors and the prediction of associated diseases. Future studies exploring the potential correlation between mediastinal seminoma and azoospermia will be prospective.

## Data availability statement

The original contributions presented in the study are included in the article/supplementary material. Further inquiries can be directed to the corresponding author.

## Ethics statement

The studies involving humans were approved by the Ethics Committee of Suzhou Kowloon Hospital. The studies were conducted in accordance with the local legislation and institutional requirements. Written informed consent for participation in this study was provided by the participants’ legal guardians/next of kin. Written informed consent was obtained from the individual(s), and minor(s)’ legal guardian/next of kin, for the publication of any potentially identifiable images or data included in this article.

## Author contributions

ZL: Writing – review & editing, Writing – original draft, Supervision, Investigation, Data curation, Conceptualization. QZ: Writing – original draft, Methodology. SN: Writing – original draft. KX: Writing – review & editing. ZX: Writing – original draft. PY: Writing – review & editing, Writing – original draft, Supervision, Conceptualization.

## References

[1] Woolner LB Jamplis RW Kirklin JW . Seminoma (germinoma) apparently primary in the anterior mediastinum. N Engl J Med. (1955) 252:653–7. doi: 10.1056/NEJM195504212521602 14370402

[2] Napieralska A Majewski W Osewski W Miszczyk L . Primary mediastinal seminoma. J Thorac Dis. (2018) 10:4335–41. doi: 10.21037/jtd.2018.06.120 PMC6105968 30174881

[3] Ozgun G Nappi L . Primary mediastinal germ cell tumors: A thorough literature review. Biomedicines. (2023) 11:487. doi: 10.3390/biomedicines11020487 36831022 PMC9953372

[4] El-Zaatari ZM Ro JY . Mediastinal germ cell tumors: A review and update on pathologic, clinical, and molecular features. Adv Anat Pathol. (2021) 28:335–50. doi: 10.1097/PAP.0000000000000304 34029275

[5] Xiu W Pang J Hu Y Shi H . Immune-related mechanisms and immunotherapy in extragonadal germ cell tumors. Front Immunol. (2023) 14:1145788. doi: 10.3389/fimmu.2023.1145788 37138865 PMC10149945

[6] Ronchi A Cozzolino I Montella M Panarese I Zito Marino F Rossetti S . Extragonadal germ cell tumors: Not just a matter of location. A review about clinical, molecular and pathological features. Cancer Med. (2019) 8:6832–40. doi: 10.1002/cam4.2195 PMC6853824 31568647

[7] De Felici M Klinger FG Campolo F Balistreri CR Barchi M Dolci S . To be or not to be a germ cell: the extragonadal germ cell tumor paradigm. Int J Mol Sci. (2021) 22:5982. doi: 10.3390/ijms22115982 34205983 PMC8199495

[8] Guida E Tassinari V Colopi A Todaro F Cesarini V Jannini B . MAPK activation drives male and female mouse teratocarcinomas from late primordial germ cells. J Cell Sci. (2022) 135(8):jcs259375. doi: 10.1242/jcs.259375 35297490

[9] Bokemeyer C Nichols CR Droz JP Schmoll HJ Horwich A Gerl A . Extragonadal germ cell tumors of the mediastinum and retroperitoneum: results from an international analysis. J Clin Oncol. (2002) 20(7):1864–73. doi: 10.1200/JCO.2002.07.062 11919246

[10] Resti G Secondino S Necchi A Fornarini G Pedrazzoli P . Primary mediastinal germ cell tumors. Semin Oncol. (2019) 46:107–11. doi: 10.1053/j.seminoncol.2019.04.001 31076171

[11] Wang L Zhao J An T Wang Y Zhuo M Wu M . Clinical characteristics and outcomes of patients with primary mediastinal germ cell tumors: A single-center experience. Front Oncol. (2020) 10:1137. doi: 10.3389/fonc.2020.01137 32766147 PMC7378816

[12] Nichols CR . Mediastinal germ cell tumors. Clinical features and biologic correlates. Chest. (1991) 99:472–9. doi: 10.1378/chest.99.2.472 1846573

[13] Albany C Einhorn LH . Extragonadal germ cell tumors: clinical presentation and management. Curr Opin Oncol. (2013) 25:261–5. doi: 10.1097/CCO.0b013e32835f085d 23422328

[14] AACR Cancer Progress Report 2022 Steering Committee . Cancer in 2022. Cancer Discovery. (2022) 12:2733–8. doi: 10.1158/2159-8290.CD-22-1134 36458429

[15] Gilligan TD Seidenfeld J Basch EM Einhorn LH Fancher T Smith DC . American Society of Clinical Oncology. American Society of Clinical Oncology Clinical Practice Guideline on uses of serum tumor markers in adult males with germ cell tumors. J Clin Oncol. (2010) 28(20):3388–404. doi: 10.1200/JCO.2009.26.4481 20530278

[16] Fischer A Richter A Filmar S Kircher S Rosenwald A Küffer S . Primary mediastinal germ cell tumours: an immunohistochemical and molecular diagnostic approach. Histopathology. (2022) 80(2):381–96. doi: 10.1111/his.14560 34506648

[17] Liu A Cheng L Du J Peng Y Allan RW Wei L . Diagnostic utility of novel stem cell markers SALL4, OCT4, NANOG, SOX2, UTF1, and TCL1 in primary mediastinal germ cell tumors. Am J Surg Pathol. (2010) 34:697–706. doi: 10.1097/PAS.0b013e3181db84aa 20410807

[18] Weissferdt A Rodriguez-Canales J Liu H Fujimoto J Wistuba II Moran CA . Primary mediastinal seminomas: a comprehensive immunohistochemical study with a focus on novel markers. Hum Pathol. (2015) 46:376–83. doi: 10.1016/j.humpath.2014.11.009 25576290

[19] Zhai Y Chen B Feng X Liu K Wang S Hui Z . Chemoradiotherapy is an alternative choice for patients with primary mediastinal seminoma. Radiat Oncol. (2022) 17(1):58. doi: 10.1186/s13014-022-02013-6 35346279 PMC8961932

[20] Childs WJ Goldstraw P Nicholls JE Dearnaley DP Horwich A . Primary Malignant mediastinal germ cell tumours: improved prognosis with platinum-based chemotherapy and surgery. Br J Cancer. (1993) 67:1098–101. doi: 10.1038/bjc.1993.201 PMC1968447 8494705

[21] Koizumi T Kanda S Nihonmatu R Gomi D Sekiguchi N Noguchi T . Primary mediastinal germ cell tumors - A retrospective analysis of >30 years of experience in a single institution. Thorac Cancer. (2021) 12:807–13. doi: 10.1111/1759-7714.13859 PMC7952792 33502089

[22] Bokemeyer C Droz JP Horwich A Gerl A Fossa SD Beyer J . Extragonadal seminoma: an international multicenter analysis of prognostic factors and long term treatment outcome. Cancer. (2001) 91(7):1394–401. doi: 10.1002/(ISSN)1097-0142 11283942

[23] Sigg S Heidenreich A Papachristofilou A Fankhauser CD . How much chemotherapy is required to optimise long-term outcomes in clinical stage 2 seminoma? Eur Urol. (2023) 84(1):32–5. doi: 10.1016/j.eururo.2022.12.010 36609008

[24] Minhas S Bettocchi C Boeri L Capogrosso P Carvalho J Cilesiz NC . EAU working group on male sexual and reproductive health. European association of urology guidelines on male sexual and reproductive health: 2021 update on male infertility. Eur Urol. (2021) 80(5):603–20. doi: 10.1016/j.eururo.2021.08.014 34511305

[25] Cioppi F Rosta V Krausz C . Genetics of Azoospermia. Int J Mol Sci. (2021) 22:3264. doi: 10.3390/ijms22063264 33806855 PMC8004677

[26] Jiang H Zhang Y Ma H Fan S Zhang H Shi Q . Identification of pathogenic mutations from nonobstructive azoospermia patients. Biol Reprod. (2022) 107:85–94. doi: 10.1093/biolre/ioac089 35532179

[27] Mazelli R Rucci C Vaiarelli A Cimadomo D Ubaldi FM Foresta C . Male factor infertility and assisted reproductive technologies: indications, minimum access criteria and outcomes. J Endocrinol Invest. (2023) 46(6):1079–85. doi: 10.1007/s40618-022-02000-4 PMC10185595 36633791

[28] Esteves SC Achermann APP Simoni M Santi D Casarini L . Male infertility and gonadotropin treatment: What can we learn from real-world data? Best Pract Res Clin Obstet Gynaecol. (2023) 86:102310. doi: 10.1016/j.bpobgyn.2022.102310 36682942

[29] Eisenberg ML Betts P Herder D Lamb DJ Lipshultz LI . Increased risk of cancer among azoospermic men. Fertil Steril. (2013) 100:681–5. doi: 10.1016/j.fertnstert.2013.05.022 PMC3759541 23790640

[30] Glazer CH Eisenberg ML Tøttenborg SS Giwercman A Flachs EM Bräuner EV . Male factor infertility and risk of death: a nationwide record-linkage study. Hum Reprod. (2019) 34(11):2266–73. doi: 10.1093/humrep/dez189 31725880

[31] Yumura Y Takeshima T Komeya M Karibe J Kuroda S Saito T . Long-term fertility function sequelae in young male cancer survivors. World J Mens Health. (2023) 41(2):255–71. doi: 10.5534/wjmh.220102 PMC10042651 36593712

[32] Heidenreich A Srivastava S Moul JW Hofmann R . Molecular genetic parameters in pathogenesis and prognosis of testicular germ cell tumors. Eur Urol. (2000) 37:121–35. doi: 10.1159/000020128 10705188

[33] de Vries G Rosas-Plaza X van Vugt MATM Gietema JA de Jong S . Testicular cancer: Determinants of cisplatin sensitivity and novel therapeutic opportunities. Cancer Treat Rev. (2020) 88:102054. doi: 10.1016/j.ctrv.2020.102054 32593915

[34] Batool A Karimi N Wu XN Chen SR Liu YX . Testicular germ cell tumor: a comprehensive review. Cell Mol Life Sci. (2019) 76(9):1713–27. doi: 10.1007/s00018-019-03022-7 PMC11105513 30671589

