# Supplementary figures and images for: Primary mediastinal seminoma with azoospermia: case report and review of the literature
Source: Front Oncol. 2024 May 17;14:1309803. doi: 10.3389/fonc.2024.1309803 (PMC11140109; doi:10.3389/fonc.2024.1309803)

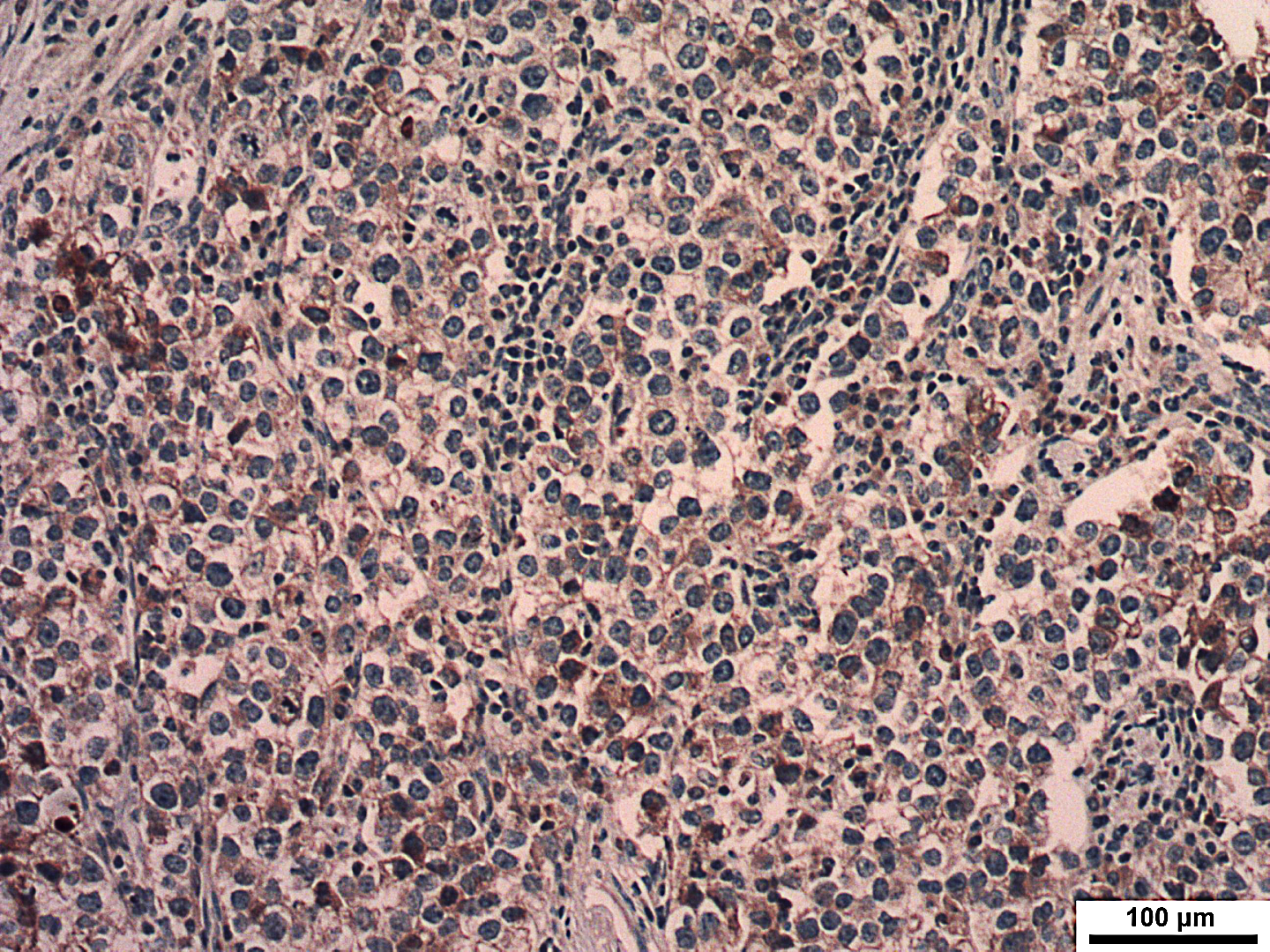

Supplement: Supplementary file 2 [file DataSheet_2.zip › supplementary figure 4A-F/AFP Fig.4C.tiff]

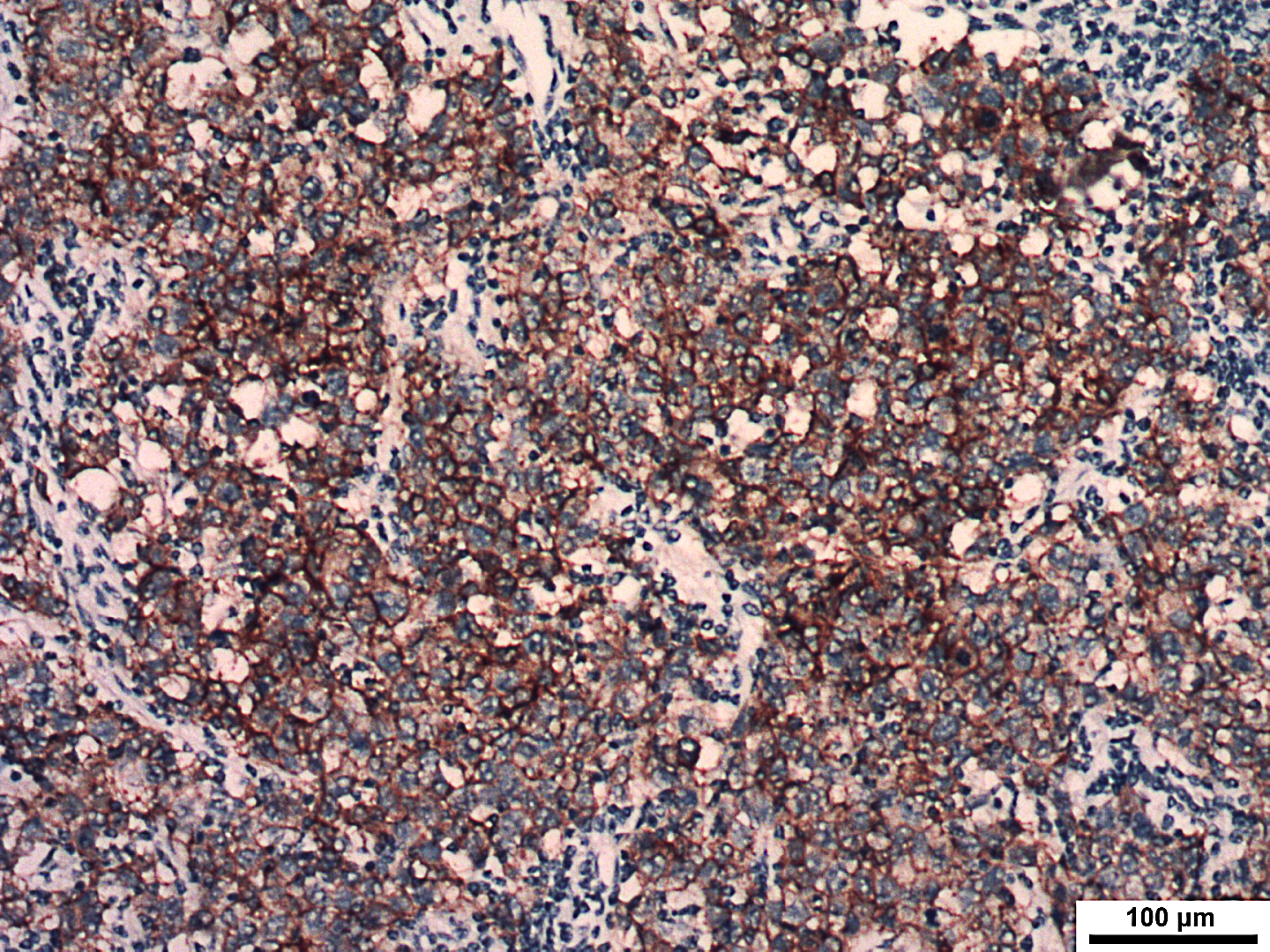

Supplement: Supplementary file 2 [file DataSheet_2.zip › supplementary figure 4A-F/CD117 Fig.4D.tiff]

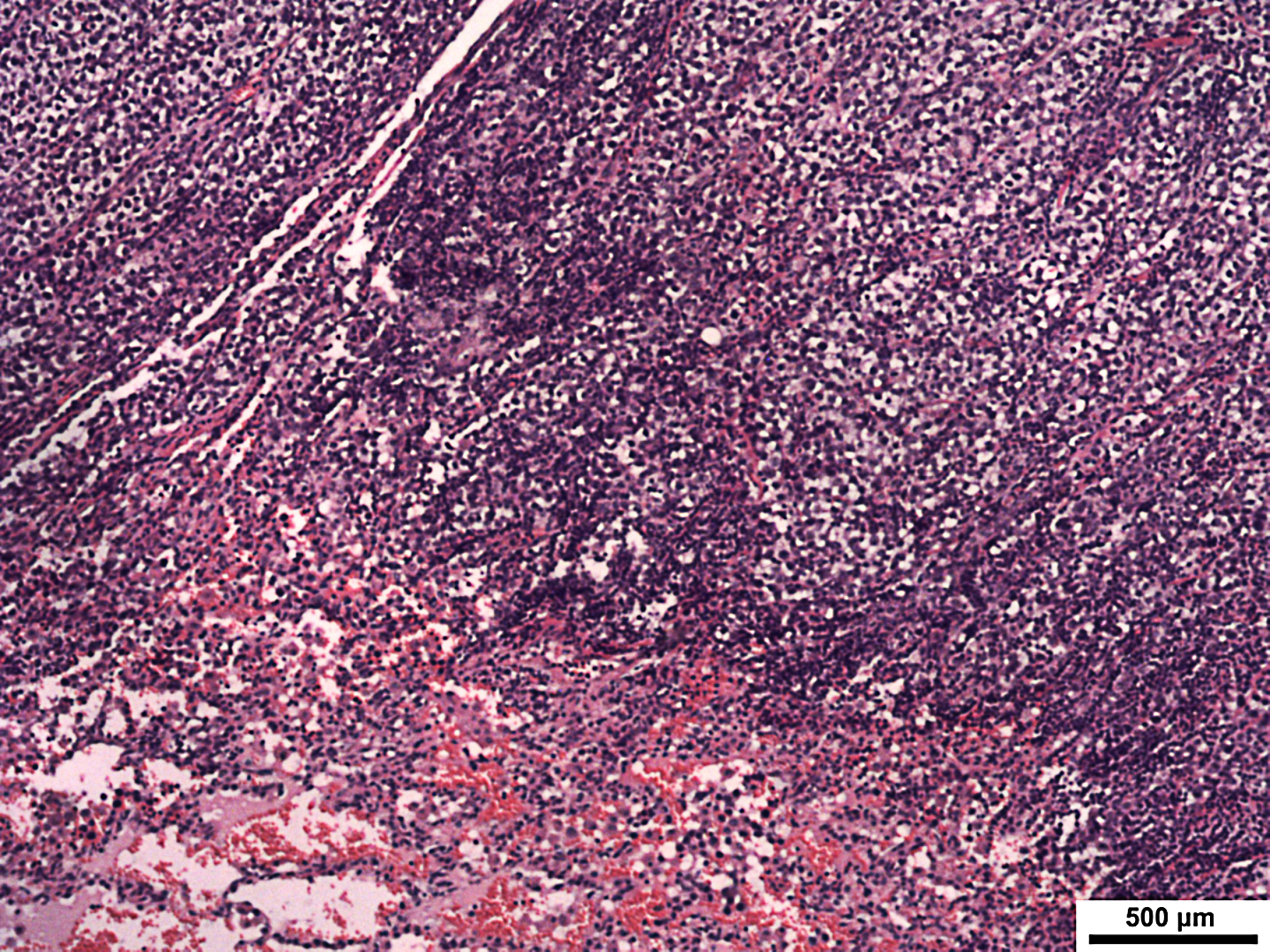

Supplement: Supplementary file 2 [file DataSheet_2.zip › supplementary figure 4A-F/HE Fig.4A.tiff]

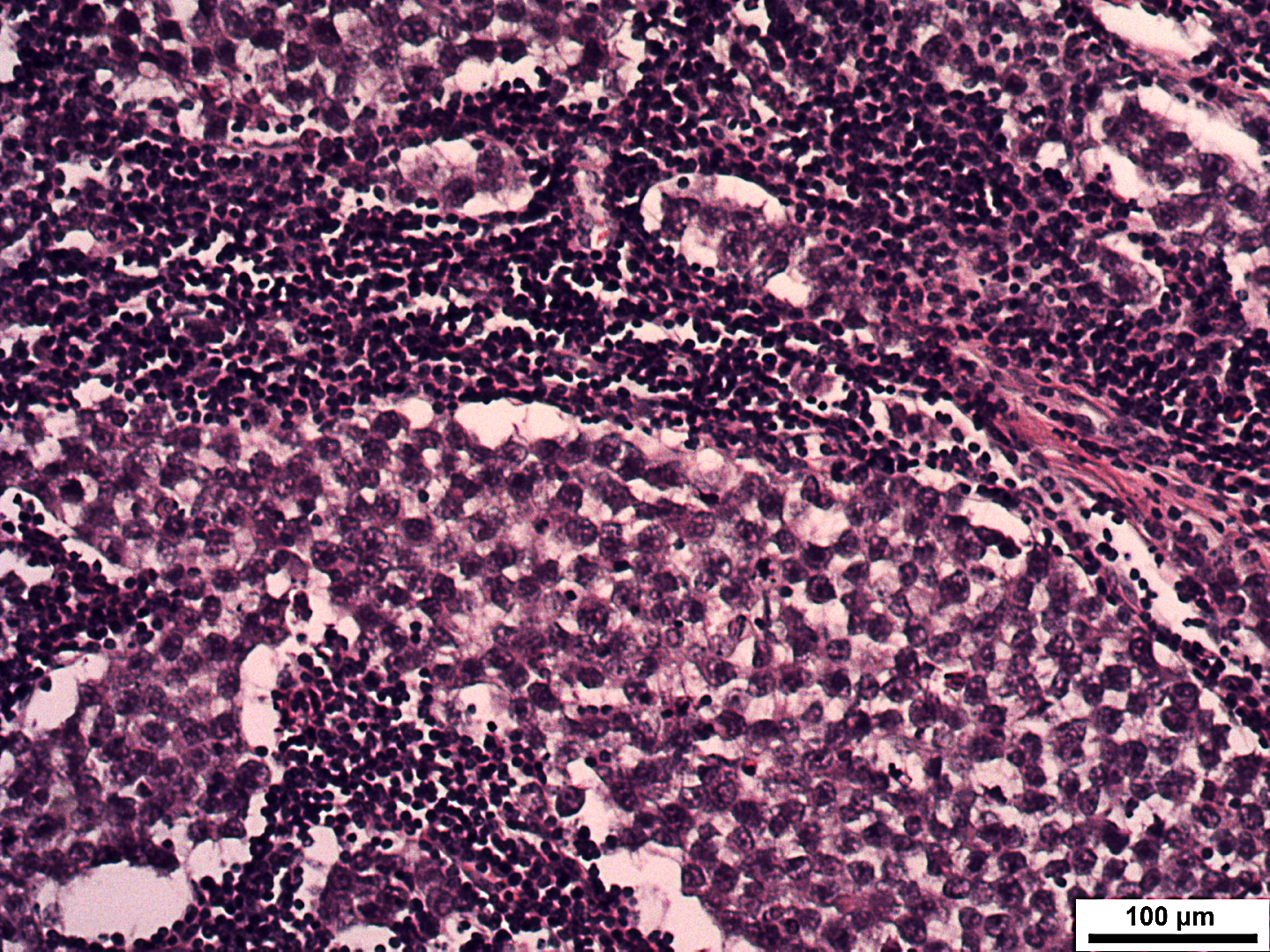

Supplement: Supplementary file 2 [file DataSheet_2.zip › supplementary figure 4A-F/HE Fig.4B.tiff]

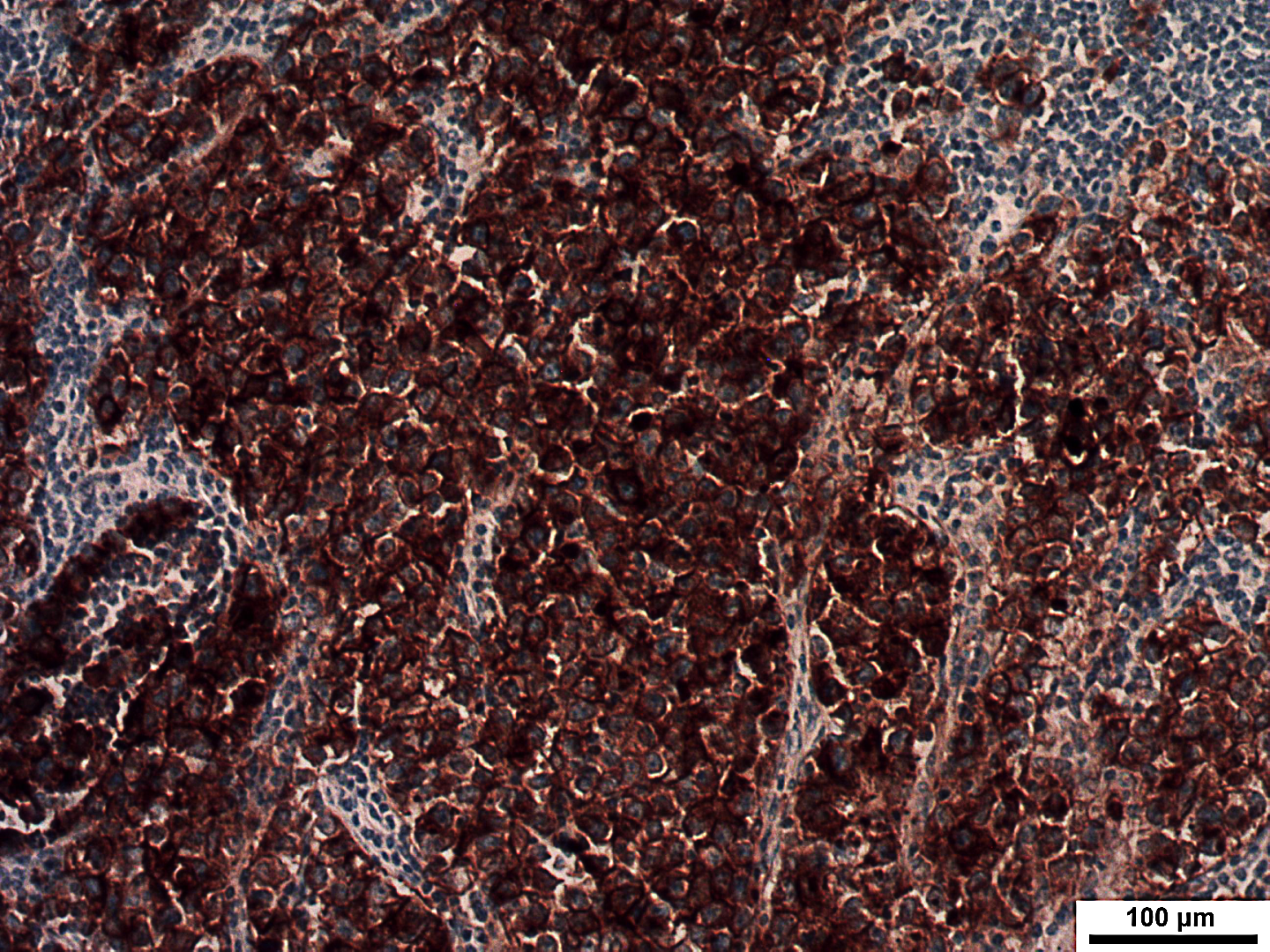

Supplement: Supplementary file 2 [file DataSheet_2.zip › supplementary figure 4A-F/PLAP Fig.4F.tiff]

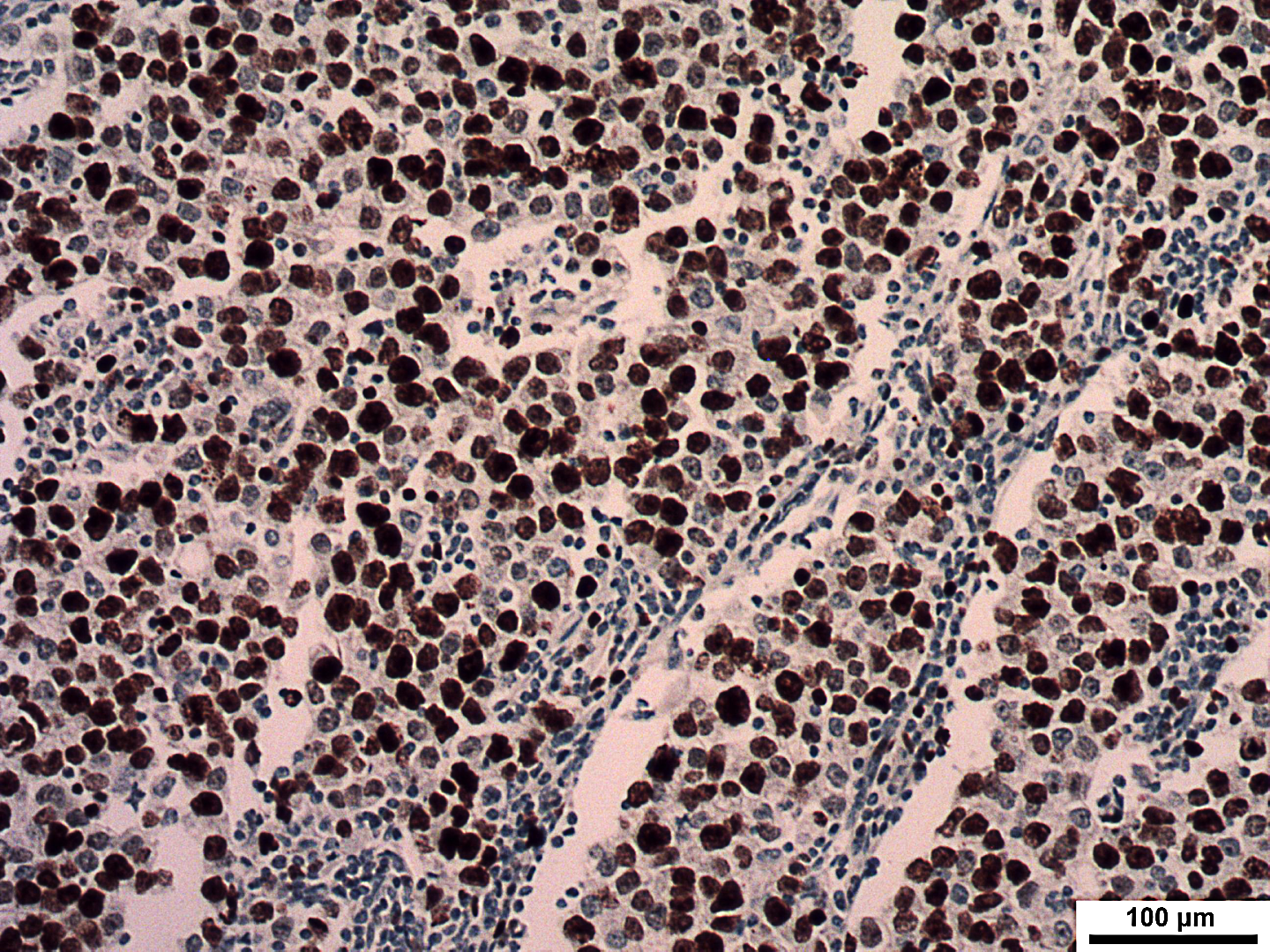

Supplement: Supplementary file 2 [file DataSheet_2.zip › supplementary figure 4A-F/ki-67 Fig.4E.tiff]

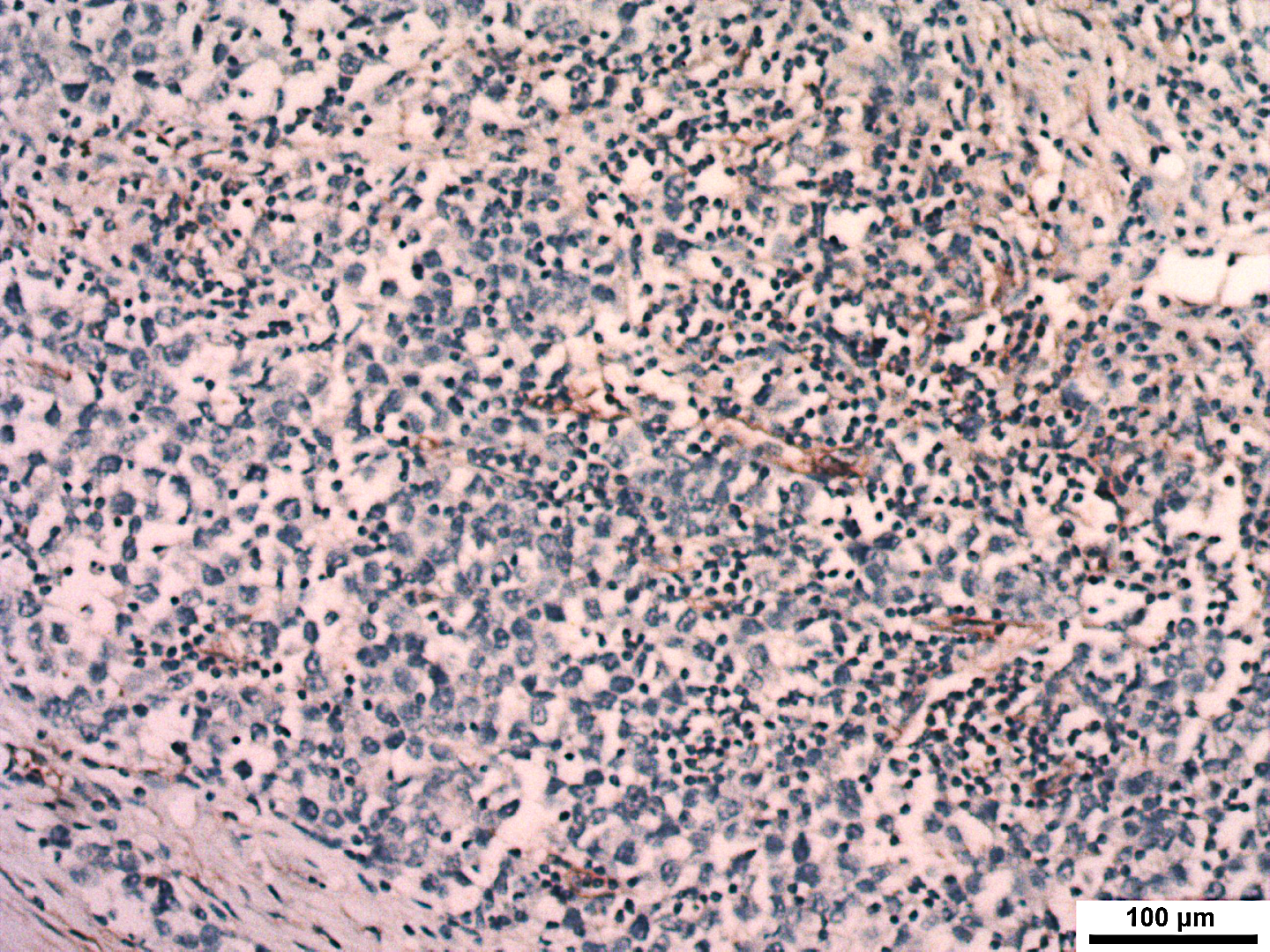

Supplement: Supplementary file 3 [file DataSheet_3.zip › supplementary figure 4G-I/CD99 Fig.4G.tiff]

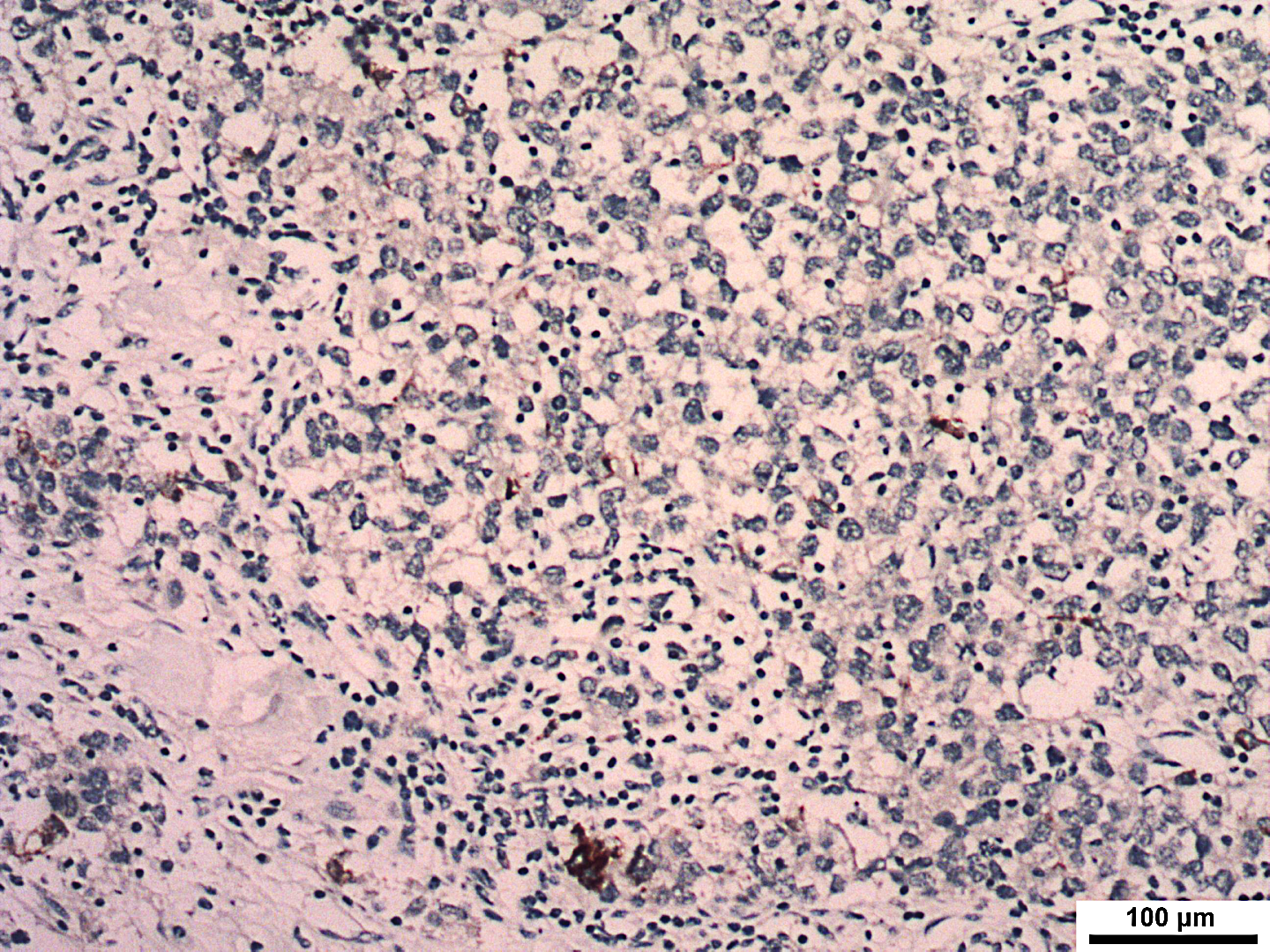

Supplement: Supplementary file 3 [file DataSheet_3.zip › supplementary figure 4G-I/CK-pan Fig.4H.tiff]

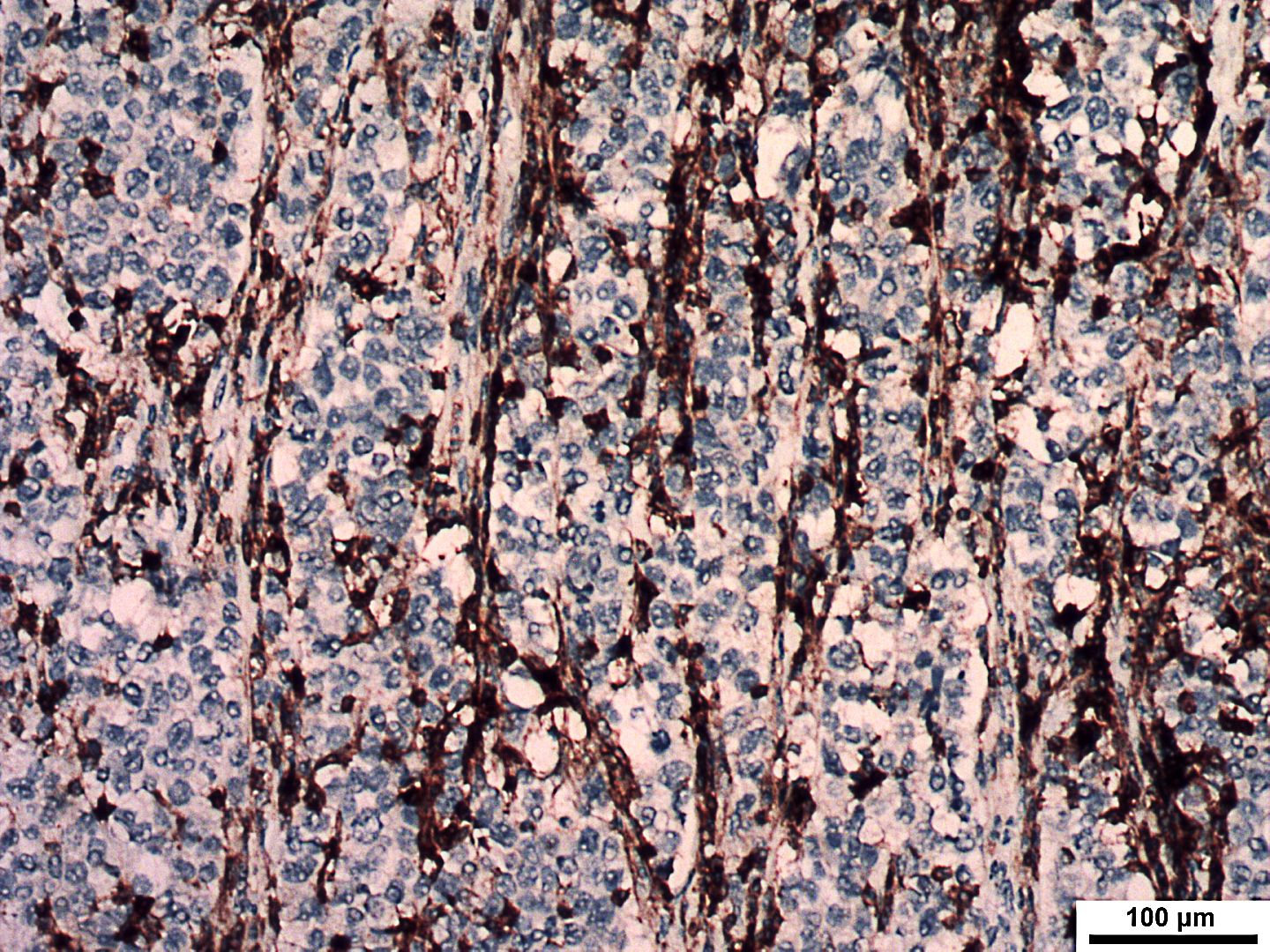

Supplement: Supplementary file 3 [file DataSheet_3.zip › supplementary figure 4G-I/LCA Fig.4I.tiff]
